# Recent Advances in Understanding Mammalian Prion Structure: A Mini Review

**DOI:** 10.3389/fnmol.2019.00169

**Published:** 2019-07-09

**Authors:** Cassandra Terry, Jonathan D. F. Wadsworth

**Affiliations:** ^1^Molecular Systems for Health Research Group, School of Human Sciences, London Metropolitan University, London, United Kingdom; ^2^MRC Prion Unit at UCL, UCL Institute of Prion Diseases, University College London, London, United Kingdom

**Keywords:** Alzheimer’s disease, prion disease, prion, prion-like, prion structure, amyloid beta, α-synuclein, tau

## Abstract

Prions are lethal pathogens, which cause fatal neurodegenerative diseases in mammals. They are unique infectious agents and are composed of self-propagating multi-chain assemblies of misfolded host-encoded prion protein (PrP). Understanding prion structure is fundamental to understanding prion disease pathogenesis however to date, the high-resolution structure of authentic *ex vivo* infectious prions remains unknown. Advances in determining prion structure have been severely impeded by the difficulty in recovering relatively homogeneous prion particles from infected brain and definitively associating infectivity with the PrP assembly state. Recently, however, images of highly infectious *ex vivo* PrP rods that produce prion-strain specific disease phenotypes in mice have been obtained using cryo-electron microscopy and atomic force microscopy. These images have provided the most detailed description of *ex vivo* mammalian prions reported to date and have established that prions isolated from multiple strains have a common hierarchical structure. Misfolded PrP is assembled into 20 nm wide rods containing two fibers, each with double helical repeating substructure, separated by a characteristic central gap 8–10 nm in width. Irregularly structured material with adhesive properties distinct to that of the fibers is present within the central gap of the rod. Prions are clearly distinguishable from non-infectious recombinant PrP fibrils generated *in vitro* and from all other propagating protein structures so far described in other neurodegenerative diseases. The basic architecture of mammalian prions appears to be exceptional and fundamental to their lethal pathogenicity.

## Introduction

Prion diseases are a closely related group of neurodegenerative conditions which affect both humans and animals. They include bovine spongiform encephalopathy (BSE) in cattle, scrapie in sheep and goats, chronic wasting disease (CWD) in deer and elk, and the human prion diseases, kuru, Creutzfeldt-Jakob disease (CJD), variant CJD (vCJD), fatal familial insomnia (FFI) and Gerstmann–Sträussler–Scheinker disease (GSS; Prusiner, 1998; Collinge, 2001; Wadsworth and Collinge, 2011; Haïk and Brandel, 2014; Greenlee and Greenlee, 2015; Benestad and Telling, 2018; Rossi et al., 2019). They are exceptional pathogens (devoid of significant coding nucleic acid) and comprise infectious polymeric assemblies of misfolded host-encoded prion protein (PrP; Prusiner, 1998; Collinge and Clarke, 2007; Collinge, 2016). Prions propagate by means of seeded protein polymerization, which involves recruitment of PrP monomers to fibrillar assemblies followed by fragmentation of these structures to generate more “seeds.” Different prion strains produce different disease phenotypes in the same inbred host and appear to be encoded by distinct misfolded PrP conformations and quaternary assembly states (Prusiner, 1998; Collinge and Clarke, 2007; Collinge, 2016).

While lacking the overt infectivity of prions, many other proteins are also capable of seeded protein misfolding and the generation of self-propagating polymeric or amyloid protein assemblies now appears to be widely involved in the pathogenesis of many other human diseases. Consequently “prion-like” mechanisms and the prion strain phenomena have become a major research focus in other neurodegenerative conditions, in particular, in Alzheimer’s disease (AD) and Parkinson’s disease where propagating assemblies of amyloid-β, tau and α-synuclein are being studied (Prusiner, 2013; Goedert, 2015; Collinge, 2016; Walker, 2016; Qiang et al., 2017; Condello et al., 2018; Peng et al., 2018; Vaquer-Alicea and Diamond, 2019). Notably, in the case of tau, structurally distinct fibrillar assemblies from brain have recently been characterized in AD, Pick’s disease and chronic traumatic encephalopathy (CTE) strongly suggesting that distinct strains of propagating tau assemblies are contributing to different disease phenotypes in humans (Fitzpatrick et al., 2017; Falcon et al., 2018; Falcon et al., 2019).

Significantly, while iatrogenic transmission of neurodegenerative diseases was thought to be restricted to prions, there is now considerable evidence for human transmission of cerebral amyloid angiopathy and amyloid-β protein pathology resulting from discontinued medical practices involving treatment with human cadaveric pituitary-derived growth hormone or cadaveric dura mater grafting (Jaunmuktane et al., 2015; Frontzek et al., 2016; Ritchie et al., 2017; Cali et al., 2018; Purro et al., 2018; Banerjee et al., 2019). These findings now underscore the importance of fully understanding the prion-like properties of proteopathic seeds generated in other neurodegenerative diseases and systematically establishing their potential risks for iatrogenic transmission.

At present there is considerable debate regarding the nomenclature that should be used in describing the propagation of non-PrP protein assemblies (to distinguish them from lethal PrP prions) with terms such as propagons and prionoids being proposed (Collinge, 2016; Kara et al., 2018; Scheckel and Aguzzi, 2018; Duyckaerts et al., 2019; Eraña, 2019). Biological criteria that a propagating protein assembly must fulfill in order to be regarded as truly “prion-like” have yet to be defined (Kara et al., 2018; Scheckel and Aguzzi, 2018; Duyckaerts et al., 2019; Eraña, 2019) and structural classification of propagating protein assemblies remains a key goal. Indeed, in this context, it should be noted that propagation and spread of assemblies of amyloid-β, tau and α-synuclein in animal models of other neurodegenerative diseases rarely result in lethal neurodegeneration, suggesting that the basic architecture of mammalian prions may be unique and central to their lethality (Collinge, 2016; Terry et al., 2019).

Here, we now highlight recent advances in prion isolation and structural characterization that have provided the first meaningful opportunity to compare the basic architecture of authentic infectious mammalian prions with the structures of protein assemblies from other neurodegenerative diseases. Current available data indicate that mammalian prions have unique structural features that readily distinguish them from propagating assemblies of amyloid-β, tau and α-synuclein that have been described in other neurodegenerative diseases.

## Key Molecular Features of Prion Diseases

The central feature of prion diseases is the aberrant misfolding of PrP which can adopt distinct conformations and assembly states (Prusiner, 1998; Collinge and Clarke, 2007; Rodriguez et al., 2017). The normal form of the protein, referred to as PrP^C^ (the cellular isoform) is a highly conserved cell surface glycosylphosphatidylinositol (GPI)-anchored sialoglycoprotein with an ordered C-terminal domain containing three α-helices, a short anti-parallel β-sheet and a flexible disordered N-terminal domain (Wüthrich and Riek, 2001; Rodriguez et al., 2017) and is soluble in detergents and sensitive to digestion with proteases. In contrast, disease-associated isoforms of PrP that comprise infectious prion assemblies are found only in prion-infected tissue and are composed of detergent-insoluble polymeric PrP structures some of which become protease-resistant and are classically termed PrP^Sc^ (the scrapie isoform; Meyer et al., 1986; Prusiner, 1987, 1991, 1998). PrP^Sc^ is derived from PrP^C^ by conformational rearrangement and neither systematic study of known covalent post-translational modifications nor amino acid sequencing have shown any consistent variations between PrP^Sc^ and PrP^C^ (Prusiner, 1991, 1998; Riesner, 2003). The structural transition of PrP^C^ to fibrillar assemblies of PrP^Sc^ involves acquisition of a beta sheet rich configuration (Pan et al., 1993; Caughey et al., 1998; Prusiner, 1998; Riesner, 2003; Rodriguez et al., 2017) likely to be similar to amyloid where beta sheets stack perpendicular to the fiber axis forming a cross-beta structure (Eisenberg and Sawaya, 2017; Iadanza et al., 2018).

To date, the critical molecular events during infection that generate prototypical PrP^Sc^ and how this causes neurodegeneration remains poorly defined. Notably, in many prion strain/host combinations the majority of disease-related PrP and prion titre is destroyed by protease-treatments that are typically used to detect prototypical PrP^Sc^ (Safar et al., 1998, 2005; Cronier et al., 2008; D’Castro et al., 2010; Tixador et al., 2010; Sandberg et al., 2014). These findings indicate that the term PrP^Sc^, often used interchangeably with prion infectivity, should be restricted to material as classically biochemically defined (infectious detergent-insoluble, protease-resistant PrP assemblies). Such prototypical PrP^Sc^ comprises a small proportion of total disease-related PrP isoforms and while it is clearly an infectious structure its specific contribution to other aspects of disease pathogenesis remains unclear (Sandberg et al., 2014). Notably in this context, it is now thought that a distinct oligomeric or monomeric PrP isoform designated PrP^L^ (for lethal) may comprise the neurotoxic species, and that prototypical PrP^Sc^ and indeed prions *per se* may not themselves be highly neurotoxic (Collinge and Clarke, 2007; Sandberg et al., 2011, 2014; Collinge, 2016). Determining the structural relationship between infectious and neurotoxic PrP species and whether protease-sensitive and protease-resistant infectious PrP assemblies are simply different-sized particles of essentially the same PrP structure has yet to be resolved. Consequently, it is now clear that a complete understanding of prion disease pathogenesis will require knowledge not only of infectious PrP structures but also the role of other PrP assemblies that may be variably generated during prion disease pathogenesis (Collinge and Clarke, 2007; Collinge, 2016).

## Brief Overview of Historical Studies on Prion Structure

High resolution structural analysis of infectious mammalian prions has been obstructed by two central problems. First, the difficulty in recovering relatively homogeneous particles from affected tissue whose composition and PrP assembly state can be directly correlated with infectivity, and second, the failure to reproducibly generate high-titre synthetic prions from fully defined constituents. Although the formation of prions *in vitro* from recombinant PrP or isolated PrP^C^ preparations (either alone or in combination with non-protein cofactors) has been reported, specific-infectivities are generally too low for meaningful structural analysis (Collinge and Clarke, 2007; Diaz-Espinoza and Soto, 2012; Schmidt et al., 2015; Collinge, 2016) and preparations with high prion titre (for example Moudjou et al., 2016) have not yet been structurally characterized. Consequently, the goal of solving infectious prion structure continues to rely upon the isolation of high-titre *ex vivo* prions in a form suitable for detailed structural study.

Scrapie associated fibrils (SAFs; Merz et al., 1981) and prion rods (Prusiner et al., 1983) were first described in prion-enriched isolates from infected brain tissue more than 35 years ago. While contemporary comparison of SAFs and prion rods now suggest they are synonymous, at the time of their discovery (before the PrP gene was identified) they were interpreted very differently. While Merz et al. (1984) proposed that SAFs may represent a new class of filamentous animal virus, Prusiner et al. (1983) proposed that prion rods were infectious protein assemblies (prions) composed of a protein designated PrP 27–30 (subsequently established to be proteolytically truncated PrP^Sc^) and that the morphology of the prion rods was incompatible with a uniform virus structure (DeArmond et al., 1985); in particular, that the length of the prion rods was not essential for preservation of prion infectivity (Barry et al., 1985; Prusiner, 1987). Subsequently, Prusiner (1991, 1998) proposed that the prion rods were an artifact of purification and suggested that protease-truncation of PrP^Sc^ to PrP 27–30 in the presence of detergent facilitated the assembly of prion rods from smaller infectious oligomers of PrP^Sc^ (McKinley et al., 1991). While this proposal at the time clearly excluded a viral etiology for prion diseases, this situation also left the field having to contend with the idea that large fibrillar PrP assemblies associated with prion infectivity might not actually represent authentic biologically relevant structures. Consequently, many researchers chose not to pursue structural characterization of the prion rods and instead focused on either trying to isolate smaller infectious oligomers of PrP^Sc^ from infected brain or generating synthetic prions from bacterially expressed recombinant PrP. While numerous studies have now proposed various possible PrP structures as the authentic infectious prion assembly state (Silveira et al., 2005; Sim and Caughey, 2009; Wille et al., 2009; Requena and Wille, 2014; Vázquez-Fernández et al., 2016, 2017) none of these have been convincingly correlated with high specific prion infectivity and no international consensus has been reached on their *in vivo* relevance (Baskakov et al., 2019).

## Recent Progress in Purifying and Characterizing Mammalian Prions

The availability of cell-based prion bioassays (Klöhn et al., 2003; Mahal et al., 2007; Schmidt et al., 2015) has recently enabled the development of novel procedures for isolating extremely pure, intact high-titre infectious prions from mammalian brain (Wenborn et al., 2015). Misfolded PrP in these preparations is highly protease-resistant and is assembled into rod-like structures, PrP rods (akin to prion rods identified by Prusiner and colleagues), which faithfully transmit prion strain-specific phenotypes when inoculated into mice (Wenborn et al., 2015). PrP rods are intrinsically infectious in cell culture infectivity assays and form aggregates whose size and number appear to determine the number of infectious units available to cells at inoculation (Terry et al., 2016). Filtration of the rod preparations showed no evidence for the presence of small infectious oligomers of PrP^Sc^ and thermal and chemical inactivation profiles of prion infectivity indicated the destruction of the same infectious structures in isolated PrP rod preparations or starting brain homogenate (Terry et al., 2016). Importantly, exploration of the origin of the PrP rods showed no evidence for their artifactual generation during purification as they could be isolated from brain without using detergent and the dimensions and morphology of the rods from crude brain homogenate were not noticeably influenced by variable exposure to protease and detergent (Terry et al., 2016). Differences in the length of propagating infectious PrP rods in brain (in which the ends of the rod may comprise the infectious surface) can readily explain variance in specific prion infectivity with respect to PrP monomers and also account for the widespread distribution of infectious prion particles that is seen when prion-infected brain homogenate is fractionated by sedimentation velocity ultracentrifugation (Prusiner et al., 1987).

## Infectious *ex vivo* PrP Rods Have Unique Structural Features

**Figure 1:**
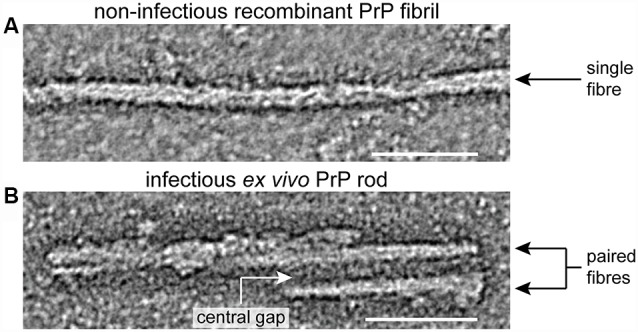
Structural differences between infectious *ex vivo* PrP rods and non-infectious recombinant prion protein (PrP) fibrils generated *in vitro*. Panels **(A,B)** show sections from negative stain electron tomography reconstructions that were originally published in (Terry et al., 2016), scale bars, 50 nm. Non-infectious recombinant PrP fibrils **(A)** appear as single fibers ~10 nm wide comprised of two closely intertwined protofilaments (Tattum et al., 2006; Terry et al., 2016). In contrast infectious *ex vivo* PrP rods **(B)** are ~20 nm wide and are composed of two fibers (each with a double helical substructure) separated by a central gap of 8–10 nm in width which is filled with irregularly structured material (Terry et al., 2016, 2019).

Examination of infectious PrP rods isolated from multiple prion strains by negative-stain electron microscopy (EM), negative-stain electron tomography, cryo-EM and atomic force microscopy (AFM) have recently revealed a common three-dimensional architecture (Terry et al., 2016, 2019). Infectious PrP rods are ~20 nm wide and are composed of two fibers (each with a double helical substructure) separated by a distinct gap of 8–10 nm in width. AFM showed that the central gap contains irregularly-structured material that appears to be compositionally distinct from the surface of the individual fibers. This finding is consistent with the idea that PrP N-linked glycans in the gap may be contributing to the overall stability of the rod and as a consequence its infectivity (Terry et al., 2016, 2019). The overall architecture of the infectious PrP rods is very distinct to the structure of non-infectious PrP fibrils generated *in vitro* from recombinant PrP which comprise long, single fibers (10 nm wide) formed by a double helical arrangement of two protofilaments (Tattum et al., 2006; Terry et al., 2016, 2019; Figure 1).

Notably, because of their helical twist, PrP rods when imaged on a surface alternate between narrower, edge-on and wider, face-on views of the structure. Consequently, the overall architecture of this twisted assembly is often hard to distinguish in EM images, which are two-dimensional density projections. However, the twist of the paired fibers in the rod, as well as a twisted two stranded structure within each fiber, becomes apparent when the three dimensional structure of the assembly is resolved by tomography (Terry et al., 2016). Significantly, visualization of the PrP rods by tomography and the ensuing recognition of their basic architecture (Terry et al., 2016, 2019) now enables the structural features of the PrP rods to be readily seen in most published EM images of SAFs and prion rods from earlier studies, including those from CJD brain (Merz et al., 1984). Collectively, these new data overturn previous dogma and firmly establish *ex vivo* PrP rods as the authentic infectious prion assembly state that should now be targeted in future high resolution imaging studies.

At present without a high-resolution three-dimensional structure of infectious PrP rods, the detailed arrangement of secondary structure components of PrP within the fibers of the rod remains unknown. Knowledge of the structures of alternative single fiber PrP amyloid fibrils (either generated *in vitro* or isolated from mice expressing mutant PrP) cannot be applied with any certainty to the PrP rods (Terry et al., 2016, 2019). From the various PrP fibrillar structures that have been characterized to date, two major structural models for prions have been proposed; the parallel in-register intermolecular β-sheet (PIRIBS) architectures and the 4-rung beta solenoid model (Baskakov et al., 2019). Determining whether either of these models applies to infectious PrP rods is now dependent on obtaining their high resolution structure.

While strain-specific structural differences in infectious PrP rods may become apparent with future application of higher resolution imaging methods (such as cryo-tomography and subtomogram averaging) their basic architecture can now be compared with fibrillar assemblies of other proteins that propagate in other neurodegenerative diseases (Table 1). Based upon the available data the structure of infectious PrP rods can be readily distinguished from fibrillar structures of tau, amyloid-β, α-synuclein, TDP-43 and SOD1 because none of these possess a prominent central gap region that resembles the PrP rods.

**Table 1:** Studies reporting the structures of fibrillar protein assemblies from patients with various neurodegenerative diseases other than prion diseases.

Neurodegenerative disease (assembled protein)	Structural method	Tissue source	Reference
Alzheimer’s disease (AD; Amyloid-β)	Negative stain EM	Brain from patients with AD	Paravastu et al. (2009)
Alzheimer’s disease (Amyloid-β)	Negative stain EM	Brain from patients with AD	Lu et al. (2013)
Alzheimer’s disease (Tau)	Cryo-EM	Brain from patients with AD	Fitzpatrick et al. (2017)
Pick’s disease (Tau)	Cryo-EM	Brain from patients with Pick’s disease	Falcon et al. (2018)
Chronic traumatic encephalopathy (CTE; Tau)	Cryo-EM	Brain from patients with CTE	Falcon et al. (2019)
Parkinson’s disease/dementia with Lewy bodies (α-synuclein)	Negative stain EM	Brain from patients with dementia with Lewy bodies	Spillantini et al. (1998)
Amyotrophic lateral sclerosis (ALS; SOD1)	Negative stain EM	Spinal cord from patients with familial ALS	Kato et al. (1997, 2000)
Frontotemporal lobar degeneration (FTLD-U) and amyotrophic lateral sclerosis (TDP-43)	Negative stain EM	Brain from patients with FTLD-U and ALS	Lin and Dickson (2008)
Frontotemporal lobar degeneration (TDP-43)	Negative stain EM	Brain from patients with FTLD with TDP-43 proteinopathy	Thorpe et al. (2008)
Amyotrophic lateral sclerosis and frontotemporal lobar degeneration (TDP-43)	Negative stain EM	Brain from patients with ALS and FTLD-with TDP-43 proteinopathy	Nonaka et al. (2013)
Frontotemporal lobar degeneration (TDP-43)	Negative stain EM	Brain from patients with FTLD with TDP-43 proteinopathy	Laferrière et al. (2019)

## Co-Propagation of Infectious PrP Rods and Transmissible PrP Amyloid

Prion-infected transgenic mice expressing mutant GPI-anchorless PrP replicate authentic prions (that are transmissible to wild-type mice) but they also develop intense PrP amyloid plaques in their brain which are not seen in the brain of prion-infected wild-type mice (Chesebro et al., 2005, 2010). Based upon the currently available evidence (summarized in Terry et al., 2019) it appears that prion infection in these mice leads to the propagation of infectious PrP rods which account for the transmissible prion infectivity and structurally distinct single PrP fibers (10 nm wide; Vázquez-Fernández et al., 2016) which account for the striking PrP amyloid plaque deposits that distinguish these mice.

Importantly, such co-propagation of infectious paired-fiber PrP rods and distinct single-fiber amyloid PrP assemblies may also be occurring in some inherited prion diseases in particular in patients with GSS disease phenotypes in which amyloid plaques are a prominent neuropathological feature (see Terry et al., 2019). This could readily explain why biochemically-distinct PrP assemblies from GSS patients with the P102L PrP mutation can transmit different phenotypes to experimental mice resulting in either a clinically silent PrP amyloidosis or a lethal spongiform encephalopathy (Piccardo et al., 2007; Barron et al., 2016; Barron, 2017). Variation in the substructure of infectious PrP rods or distinct amyloid PrP fibrils (governed by the specific PrP missense mutation) would be expected to dictate highly specific strain transmission properties *via* conformational selection (Collinge, 1999, 2016; Collinge and Clarke, 2007; Wadsworth et al., 2010) as has recently been demonstrated for *PRNP* P102L and A117V mutations (Asante et al., 2009, 2013, 2015). Notably, while patients with different *PRNP* missense mutations that produce full-length mutant PrP (for example P102L) would be expected to be capable of co-propagating both authentic prions (infectious PrP rods) and alternative transmissible PrP amyloid assemblies, patients with different *PRNP* stop mutations which produce C-terminally truncated PrP devoid of N-linked glycans (for example Y163X; Mead et al., 2013) may only be capable of propagating transmissible PrP amyloids giving rise to distinct disease phenotypes (Mead and Reilly, 2015).

## Concluding Remarks

Prion-infected brain contains multiple disease-related PrP assemblies. Infectious PrP rods comprise authentic prions that generate a lethal transmissible spongiform encephalopathy when inoculated into a susceptible host. Neurotoxicity accompanying the propagation of authentic prions is thought to involve the generation of a distinct toxic PrP species whose steady-state level may determine the rate of neurodegeneration. In some inherited prion diseases transmissible PrP amyloids only may be generated while in others transmissible PrP amyloids may variably co-propagate with authentic prions and act as a major modifier of clinicopathological phenotype. Studying the transmission properties of synthetic prion preparations is complex and experiments should be carefully designed and interpreted in order to differentiate between the generation of authentic lethal prions and transmissible PrP amyloids. At present, propagating fibrillar assemblies of proteins in other neurodegenerative diseases appear to have biological and structural properties that are more closely aligned with transmissible PrP amyloids rather than authentic lethal prions.

## Author Contributions

CT and JW wrote the article.

## Conflict of Interest Statement

JW is a shareholder of D-Gen Limited, an academic spin-out company working in the field of prion disease diagnosis, decontamination, and therapeutics. The remaining author declares that the research was conducted in the absence of any commercial or financial relationships that could be construed as a potential conflict of interest.
